# Measuring contraceptive self-efficacy in sub-Saharan Africa: development and validation of the CSESSA scale in Kenya and Nigeria^☆^^☆☆^

**DOI:** 10.1016/j.conx.2020.100041

**Published:** 2020-10-09

**Authors:** Lillian Whiting-Collins, Lindsay Grenier, Peter J. Winch, Amy Tsui, Pamela K. Donohue

**Affiliations:** a Johns Hopkins Bloomberg School of Public Health, Department of Population, Family, and Reproductive Health, 615 N. Wolfe Street, Baltimore, MD 21205, USA; b Jhpiego, 1615 Thames Street, Baltimore, MD 21231, USA; c Johns Hopkins Bloomberg School of Public Health, Department of International Health, 615 N. Wolfe Street, Baltimore, MD 21205, USA; d Johns Hopkins University School of Medicine, Johns Hopkins Children's Center, 1800 Orleans Street, Room 8527, Baltimore, MD 21287, USA

**Keywords:** Contraception, Contraceptive self-efficacy, Sub-Saharan Africa

## Abstract

**Objectives:**

Contraceptive self-efficacy, a women's belief about her own ability to complete the actions necessary for successful family planning, is a well-documented determinant of contraceptive use. However, there is currently no validated measure appropriate for low-resource settings. We developed and tested a new scale to measure Contraceptive Self-Efficacy among women in sub-Saharan Africa (CSESSA) using samples in Kenya and Nigeria.

**Study design:**

The CSESSA scale was administered to women in Kenya (*n* = 314) and Nigeria (*n* = 414). Reliability and validity were analyzed separately by setting. Validity analysis included assessment of the area under the curve (AUC) to demonstrate predictive capability of CSESSA score for contraceptive use. Logistic regression was employed to test the relationship between CSESSA score and contraceptive use.

**Results:**

Item reduction resulted in 11 items in Kenya (*α* = 0.90) and 10 items in Nigeria (*α* = 0.93). Three domains of contraceptive self-efficacy emerged in both settings: (1) husband/partner communication, (2) provider communication and (3) choosing and managing a method. Items related to the first two subscales, but not the third, were identical across settings. The AUC indicated predictive capability as mild in Kenya (AUC = 0.58) and strong in Nigeria (AUC = 0.73). In both settings, CSESSA score was associated with use of a modern contraceptive method at 12 months postpartum.

**Conclusions:**

The CSESSA scale is a reliable and valid measure in two countries. Variation of the third subscale by site indicates that certain scale items may be more relevant in areas of low versus high contraceptive prevalence. Further research should be done to validate this subscale in other contexts.

**Implications:**

This study contributes a reliable, valid measure of contraceptive self-efficacy in two African countries. The CSESSA scale and subscales can be administered in research (for example for evaluation of interventions to increase contraceptive uptake) or in a clinical setting to inform and improve contraceptive counseling.

## 1 Introduction

Theories of self-efficacy link an individual's beliefs about their personal capabilities to their health behaviors, and evidence indicates that practices that promote self-efficacy influence behavior change [1]. Contraceptive self-efficacy (CSE) is a woman's belief in her own ability to succeed in contraceptive initiation, management and continued use. Despite being a recognized precursor to effective contraceptive uptake [[2], [3], [4], [5]], CSE is not routinely measured in low-resource settings. Most efforts to measure contraceptive-related self-efficacy have been restricted to high-resource settings [2,3].

Levinson developed a scale to measure CSE among adolescents in high-income contexts which has been validated in a variety of settings across the United States, Canada and Mexico [2,3]. Most items in Levinson's scale are not relevant to nonadolescent women in low-resource settings as they pertain to adolescent-specific subjects such as parental knowledge of contraceptive use. There are validated scales to measure self-efficacy for condom use, sexual communication and protective sexual behaviors [[6], [7], [8], [9]]. However, review of the literature revealed no appropriate standardized tool to measure CSE in low-resource settings such as sub-Saharan Africa (SSA).

A validated measure of CSE for a low-resource context would strengthen contraceptive research and could be used to develop, evaluate and improve contraceptive promotion efforts. Responses to items on a CSE scale could identify specific behaviors for which women have low self-efficacy that can be addressed by activities to promote contraceptive adoption and continuation. Exploring CSE levels over time could lend insight to factors that influence CSE and how CSE may mediate contraceptive behavior.

We developed a new scale aiming to measure Contraceptive Self-Efficacy among women in sub-Saharan Africa (CSESSA). This paper presents findings from reliability analysis and validation of the CSESSA scale in two independent samples of women in Kenya and Nigeria.

## 2 Methods

### 2.1 Scale development

We assessed the transferability of Levinson's CSE scale to the SSA context through conversations with co-investigators and reproductive health program staff in Kenya and Nigeria. Concepts underlying certain scale items, such as partner communication and seeking contraception from a health provider, were selected for inclusion based on relevance to the target population. We reviewed the literature on factors influencing contraceptive behavior in SSA and hypothesized that CSE comprised of four domains: husband/partner communication, friend/family influence, provider communication, and choosing and managing a method [[10], [11], [12]]. Findings from focus group discussions with women of reproductive age in Nigeria informed item development for these four domains. Bandura's theories on self-efficacy guided item phrasing. Iterations of scale items were reviewed and revised by the authors with in-country colleagues and remotely by a group of reproductive health experts. The resulting 21 items were pilot-tested among 8 postpartum women attending health clinics in Kenya. Revisions were made, and three double-barreled items were removed. Eighteen items remained for scale development (Appendix A).

### 2.2 Participants

The 18-item CSESSA scale was administered to women participating in a cluster randomized trial of group-based antenatal and postnatal care in Kisumu and Machakos counties, Kenya, and Nasarawa State, Nigeria. Kisumu is located in Western Kenya on Lake Victoria, and Machakos borders Nairobi. In both counties, nearly all pregnant women receive antenatal care from a skilled provider, and the modern contraceptive prevalence rate (mCPR) is higher than the national average (59% in Kisumu and 68% in Machakos compared to 53% nationally) [13]. In Nasarawa, a diverse, underresourced state in central Nigeria, 77.1% of pregnant women receive antenatal care from a skilled provider [14]. The mCPR is slightly higher in Nasarawa State compared to Nigeria overall (14.3% compared to 12.0% nationally) [14].

Inclusion criteria and study methods are detailed elsewhere [15]. The present analysis uses cross-sectional data from a survey administered at the end of the study to participants who were 12 months postpartum. To remove any potential effect of the intervention on CSE, data are constrained to participants in control facilities only.

Due to inclusion criteria of the parent study, the sample is further limited to women who attended at least one antenatal care visit before 24 weeks’ gestation, consented to participate in the study and were available for follow-up 1 year after delivery. Women whose infants died before 12 months postpartum are excluded from analysis (*n* = 1 Kenya, 20 Nigeria) under the assumption that these women may desire another pregnancy or be pregnant at the time of survey (12 months after delivering). Data collection was completed in July 2018 in Kenya and March 2018 in Nigeria.

### 2.3 Procedures

Study staff contacted participants by phone or in-person to schedule the 12-month postpartum survey. The survey was then administered to participants in their homes by research assistants (RAs) using *RedCap* mobile technology to upload data remotely from a tablet to a secure server. All questions were read aloud to participants. For the CSESSA scale, the RAs asked women to rate the certainty with which they could do each item (for example, *discuss family size with my husband/partner*). A visual analogue scale was used as an aide to describe the response options, which ranged from 0 (*cannot do at all*) to 10 (*highly certain can do*). As needed, RAs asked participants to clarify a response falling between two tick marks and recorded the value closest to the point on the line indicated by the participant.

The survey also collected information on participants' sociodemographic characteristics and contraceptive behaviors. An indicator of household wealth was generated based on methods used by the Demographic Health Survey [16]. Women were considered to be current modern contraceptive users if they responded positively to the question “Are you currently using a family planning method to prevent pregnancy?” and reported current use of condoms, oral contraceptive pills, injectables, implant, intrauterine device, emergency contraception or sterilization.

### 2.4 Sample description

The samples in both settings surpassed the size required by the ratio of subject-to-item guidelines of 10 subjects per item and demonstrated 100% response rates for each item [17]. Demographic characteristics of study participants are provided in Table 1. Differences are notable between settings. While most women in the Kenyan sample (73.6%) were Protestant and almost all (98.1%) were literate, women in the Nigerian sample were predominantly Muslim (73.9%) and just over half (56.8%) were literate. Most women in Kenya recently delivered their first or second child (59.9% vs. 46.4% in Nigeria), while over a third of the Nigerian sample recently delivered their fourth or fifth child (35.2% vs. 19.4% in Kenya). Modern contraceptive use at 12 months postpartum was high in Kenya (73.3%) and low in Nigeria (27.5%).

**Table 1:** Demographic characteristics of study participants: postpartum women in Kisumu and Machakos counties, Kenya and Nasarawa State, Nigeria

Total	Kenya *N* = 314	Nigeria *N* = 414
	Frequency (%)	Frequency (%)
**Primary language spoken**		
English	39 (12.4)	80 (19.3)
Kiswahili	103 (32.8)	-
Hausa	-	334 (80.7)
Luo	113 (36.0)	-
Kamba	56 (17.8)	-
Other	3 (1.0)	-

**Age**		
15–19	43 (13.7)	46 (11.1)
20–24	106 (33.8)	153 (37.0)
25–29	98 (31.2)	116 (28.0)
30–34	54 (17.2)	68 (16.4)
35 +	13 (4.1)	31 (7.5)

**Religion**		
Catholicism	73 (23.3)	23 (5.6)
Islam	2 (0.6)	306 (73.9)
Protestant	231 (73.6)	85 (20.5)
Traditional	6 (1.9)	-
Other	2 (0.6)	-

**Education**		
No education/primary education/Qur'anic	149 (47.5)	253 (61.1)
Secondary/postsecondary	165 (52.6)	161 (38.9)
**Literacy**		
Can't read and write	6 (1.9)	179 (43.2)
Can read and write	308 (98.1)	235 (56.8)

**Marriage**		
Never married, single/widowed	46 (14.7)	1 (0.2)
Married/cohabiting	268 (85.4)	413 (99.8)

**Parity**		
1	91 (29.0)	103 (24.9)
2	97 (30.9)	89 (21.5)
3	65 (20.7)	76 (18.4)
4	35 (11.2)	56 (13.5)
5 or more	26 (8.2)	90 (21.7)

**Mode of transport**		
Walk	124 (39.5)	182 (44.0)
Public	180 (57.3)	201 (48.5)
Personal/other	10 (3.2)	31 (7.5)

**Household wealth**		
Lowest	83 (26.4)	129 (31.2)
Low	89 (28.3)	88 (21.3)
High	85 (27.1)	102 (24.6)
Highest	57 (18.2)	95 (22.9)

**Modern contraceptive use at 12 months postpartum**		
Yes	230 (73.3)	114 (27.5)
No	84 (26.7)	300 (72.5)

### 2.5 Ethical clearance

This study was reviewed and approved by the Johns Hopkins Bloomberg School of Public Health Institutional Review Board, the Kenya Medical Research Institute Ethics Review Committee and the National Human Research Ethics Committee of Nigeria. Written informed consent was obtained from all participants prior to data collection.

### 2.6 Analysis

We conducted psychometric analyses to assess the reliability, fit and structure of the 18-item scale independently in each setting. The reliability coefficients were high in both Kenya (Cronbach's *α* = 0.93) and Nigeria (*α* = 0.97). To remove redundant and poor-fitting scale items, we assessed item–test and item–rest correlations and factor loadings derived from principle components analysis (PCA). Items having item–rest or item–test correlation < 0.60 were removed [18]. Items were retained if their greatest factor loading was > 0.60 and second highest was < 0.30 [19]. Items with uniqueness above 0.50 were removed, leaving a total of 13 items remaining for further analysis across sites (retained items presented in Table 2). Of these 13 items, 8 were consistent across sites. Some items that were retained in Kenya were removed in Nigeria (*n* = 3) or kept in Nigeria but not Kenya (*n* = 2).

**Table 2:** Retained items^a^ by domain, CSESSA, Kenya and Nigeria

Stem	Items by domain	Kenyan scale	Nigerian scale	Response options
*How certain you are that you can…*	**Husband/partner communication**			0 *Cannot do at all* to 10 *Highly certain can do*
	1. Discuss family size with my husband/partner	X	X	
	2. Discuss if and when I'd like to get pregnant again with my husband/partner	X	X	
	3. Discuss specific family planning methods with my husband/partner	X	X	
	4. Reach an agreement with my husband/partner about use of family planning that takes my desires into account	X	X	
	**Provider communication**			
	1. Bring up the topic of family planning with a health care provider	X	X	
	2. Ask a provider to clarify something they have told me about family planning if I'm not sure I understand	X	X	
	3. Tell a provider what's important to me in choosing a family planning method	X	X	
	**Choosing and managing a method**			
	1. Choose a family planning method that will work well for me	X		
	2. Obtain the method of family planning I want, if I want one	X	X	
	3. Obtain a different method of family planning if the one I want isn't available		X	
	4. Find solutions to bothersome side effects from family planning or switch methods if needed because of bothersome side effects	X		
	5. Use a family planning method according to instructions to prevent pregnancy	X		
	6. Stop using family planning and get pregnant again if/when I want to		X	

a The following items were removed during analysis: Ask my husband/partner to use a condom if I want him to; Start a family planning method if my friends and family might find out; Continue a family planning method if my friends and family found out; Ask a provider questions I have about family planning methods; Have some control over if and when I get pregnant again.

Internal consistency of the scale with 11 items in Kenya and 10 items in Nigeria was reassessed by Cronbach's *α*. PCA was then performed to determine the structure of the scale; three factors produced eigenvalues above 1 and were visible with a scree plot. Parallel analysis confirmed a three-factor composition [20]. These were extracted through exploratory factor analysis using promax rotation and retained as the following scale domains: husband/partner communication, provider communication, and choosing and managing a method.

Reliability analysis was conducted to assess the potential of subscales by domain. Items for husband/partner communication and provider communication were identical in both settings. However, items related to choosing and managing a method varied, signaling that this domain may manifest differently in areas with low and high contraceptive prevalence (see Appendix B). Mean scores for the full scale and for each potential subscale were calculated by dividing the summative score by the number of scale items, leading to a standard range of 0–10 points. Means were compared across age, education, parity and household wealth by *t* tests.

Validity of the scale was then assessed separately for both samples (see Appendix C). We calculated the area under the curve (AUC) of a receiver operating characteristic curve to assess the predictive capability of the total CSESSA score against current modern contraceptive use (criterion-related validity) [21,22]. To address imbalance in the data, we also calculated the AUC for the total score grouped into quartiles. Construct validity was assessed through logistic regression of the total CSESSA score against current modern contraceptive use [21]. Generalized estimating equation was used to account for clustering of data at the health facility level (10 clusters per site) [23]. For sensitivity analysis, we also performed multivariate logistic regression of total scores grouped into quartiles as a predictor of current modern contraceptive use. All data were analyzed in Stata15.

## 3 Results

### 3.1 Kenya

#### 3.1.1 Reliability

Reliability analysis of the 11-item scale in Kenya produced a Cronbach's *α* of 0.90 and average interitem correlation (IIC) of 0.49, signifying a reliable measure. On a single-factor solution, the item *Obtain the method of family planning I want, if I want one* loaded highest. Three potential subscales were identified based on factor loadings (organized according to domain in Appendix D). Reliability analysis for a husband/partner communication subscale returned a strong Cronbach's *α* of 0.89 and high IIC of 0.68. The reliability indicators for a provider communication subscale and a scale for choosing and managing a method were similarly strong (*α* = 0.89, IIC 0.68; *α* = 0.88, IIC 0.71, respectively).

The 11-item CSESSA scale total scores ranged from 0 to 110 points. Dividing the summative score by the number of scale items revealed an overall mean of 8.72 and standard deviation (SD) of 1.72. Percent distribution of mean scores for the Kenya sample is highly skewed. Mean responses were high across domains: 8.24 (SD 2.57) for husband/partner communication, 8.98 (SD 1.73) for provider communication, and 9.02 (SD 1.84) for choosing and managing a method. Mean scores by demographic characteristic are shown in Table 3.

**Table 3:** CSESSA mean scores, standardized to 10-point scale, by age, education, parity, facility location and household wealth^a^

	Kenya				Nigeria			
	Full CSESSA scale	Husband/partner communication	Provider communication	Choosing and managing a method	Full CSESSA scale	Husband/partner communication	Provider communication	Choosing and managing a method
**Overall mean** (SD) [interquartile range]	8.72 (1.72) [1.82]	8.24 (2.57) [2.50]	8.98 (1.73) [1.33]	9.02 (1.84) [1.25]	6.61 (2.55) [4.00]	5.53 (3.32) [5.75]	8.05 (2.34) [3.33]	6.60 (3.38) [5.67]
**Age** 15–24 25 +	8.49 (2.03)* 8.94 (1.36)	7.97 (2.90) 8.47 (2.22)	8.68 (2.02)*** 9.26 (1.38)	8.86 (1.94) 9.17 (1.74)	6.73 (2.56) 6.50 (2.54)	5.65 (3.42) 5.43 (3.23)	8.17 (2.32) 7.94 (2.36)	6.73 (3.33) 6.49 (3.44)
**Education** No education/primary education/Qur'anic Secondary/postsecondary	8.54 (1.88) 8.89 (1.55)	8.05 (2.67) 8.40 (2.48)	8.93 (1.82) 9.04 (1.65)	8.74 (2.16)** 9.27 (1.47)	6.46 (2.66) 6.85 (2.37)	5.04 (3.47)*** 6.32 (5.87)	8.09 (2.39) 7.98 (2.26)	6.72 (3.44) 6.43 (3.28)
**Parity** First birth Had previous birth	8.52 (1.83) 8.81 (1.67)	7.89 (2.92) 8.37 (2.41)	8.72 (1.83) 9.09 (1.68)	8.99 (1.61) 9.03 (1.93)	6.59 (2.60) 6.62 (2.54)	5.70 (3.25) 5.48 (3.34)	7.97 (2.50 8.07 (2.29)	6.39 (3.41) 6.68 (3.38)
**Household wealth** Lowest–Low High–Highest	8.66 (1.83) 8.86 (1.45)	8.10 (7.78) 8.51 (2.06)	9.02 (1.71) 8.91 (1.79)	8.95 (1.97) 9.16 (1.53)	6.47 (2.78) 6.77 (2.27)	5.14 (3.56)* 5.98 (2.98)	8.27 (2.52)* 7.80 (2.07)	6.43 (3.64) 6.80 (3.07)

*p < .05, **p < .01, ***p < .005.

a *t* tests assessed differences in mean score by sociodemographic characteristic, with significant results indicating a relationship between the sociodemographic characteristic and mean score.

#### 3.1.2 Validity

**Fig. 1:**
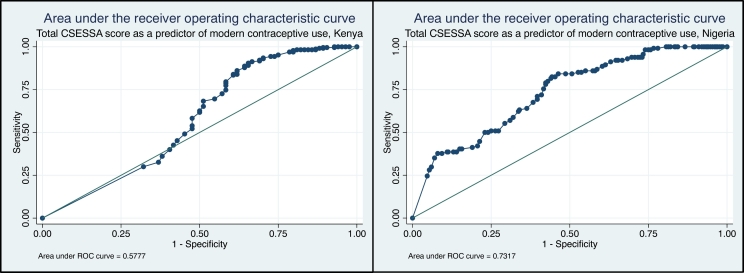
Area under the receiver operating characteristic curve for total score on the contraceptive self-efficacy in sub-Saharan Africa scale as a predictor of modern contraceptive use at 1 year postpartum, Kenya and Nigeria

**Fig. 2:**
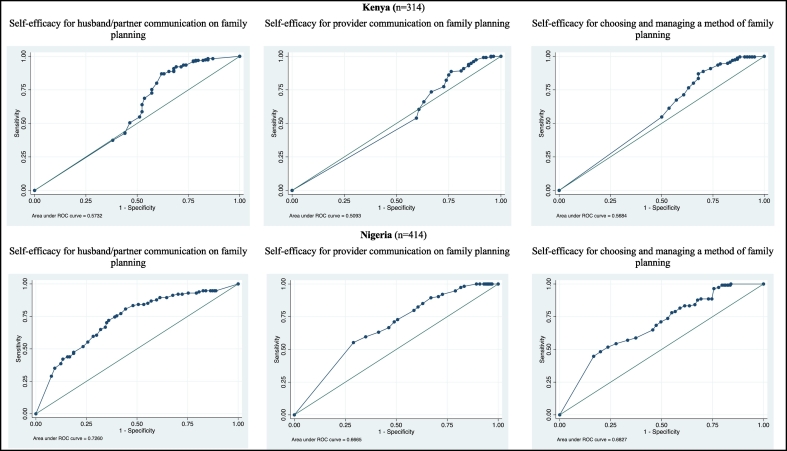
Area under the receiver operating characteristic curves for each CSESSA sub-scale as a predictor of modern contraceptive use, Kenya and Nigeria

As shown in Fig. 1, the AUC of 0.58 suggests that while a woman's total score on the CSESSA in Kenya may predict her willingness to use modern contraception, more investigation into this relationship is required. Grouping the total score into quartiles revealed an improved predictive capacity when the distribution of scores was more balanced (AUC = 0.61, standard error 0.03). Each of the three potential subscales produced a similar result with AUC ranging from 0.51 to 0.58 (Fig. 2).

Results of multivariate logistic regression indicate a valid measure: for each one-point increase in the CESSA total score, the odds of current modern contraceptive use increased by 4.0% [adjusted odds ratio (aOR) 1.04, p < .001, confidence interval (CI) 1.02–1.06] controlling for age, language, religion, education, parity and household wealth. This same relationship held when the total CSESSA score was split into quartiles (aOR 1.73, p < .001, CI 1.35–2.21), controlling for the same variables. We ran the same model to assess the relationships of each subscale to current modern contraceptive use. The total score from each subscale was significantly associated with the outcome as shown in Table 4.

**Table 4:** Multivariate logistic regressions^a^ of CSESSA total and subscale scores as predictors of current modern contraceptive use, Kenya and Nigeria

Scale	Odds ratio^b^	95% Confidence interval
**Kenya**		
Total CSESSA score (110 points)	1.04	1.02–1.06
*Total score for each subscale*		
Husband/partner communication (40 points)	1.06	1.03–1.09
Provider communication (30 points)	1.08	1.02–1.13
Choosing and managing a method (40 points)	1.09	1.05–1.13

**Nigeria**		
Total CSESSA score (100 points)	1.06	1.05–1.08
*Total score for each subscale*		
Husband/partner communication (40 points)	1.10	1.07–1.12
Provider communication (30 points)	1.18	1.11–1.25
Choosing and managing a method (30 points)	1.16	1.11–1.20

a A separate regression was run for each scale and subscale with the outcome of current modern contraceptive use; each controlled for age, language, religion, education, parity and household wealth.

b Odds ratios reflect the difference in odds of using modern contraception for each one-point increase in total score.

### 3.2 Nigeria

#### 3.2.1 Reliability

Assessment of internal consistency for the 10-item scale in Nigeria indicated strong reliability (*α* = 0.93, ICC 0.56). On a single-factor solution, the item *Discuss specific family planning methods with my husband/partner* loaded highest. Analysis of potential subscales by domain showed strong reliability but moderately high IIC. For husband/partner communication, the Cronbach's *α* was 0.94 with IIC of 0.79; provider communication returned a Cronbach's *α* of 0.95 and IIC of 0.87; and choosing and managing a method showed a Cronbach's *α* of 0.93 and IIC of 0.82. Total scores for the full 10-item scale ranged from 0 to 100. Dividing the summative score by the number of scale items revealed an overall mean of 6.61 (SD 2.55). Mean responses by domain were 5.54 (SD 3.32) for husband/partner communication, 8.05 (SD 2.34) for provider communication, and 6.60 (SD 3.38) for choosing and managing a method (Table 3).

#### 3.2.2 Validity

The AUC of 0.73 (standard error 0.03) indicates that a woman's total score on the CSESSA in Nigeria has a strong predictive capability for modern contraceptive use (Fig. 1). Grouping the total score into quartiles reduced the predictive value slightly to an AUC of 0.70 with standard error of 0.03. Similarly, all three subscales were highly predictive of current modern contraceptive use, with AUC above 0.67 (Fig. 2).

In the Nigeria sample, each one-point increase in CESSA total score (0–100) increased the odds of current modern contraceptive use by 6.0% (aOR 1.06, p < .001, CI 1.05–1.08) controlling for age, language, religion, education, parity and household wealth. In the same model, each progressive quartile of total CSESSA score increased the odds of current modern contraceptive use 3.02 times (aOR 3.02, p < .001, CI 2.23–4.09). In separate multivariate regressions, each subscale total score was statistically significantly associated with current modern contraceptive use (Table 4).

## 4 Discussion

This paper contributes a reliable, validated measure for CSE in two African countries. While the full scale provides a multifaceted and comprehensive measure of CSE, subscales allow researchers to focus on specific aspects of CSE using fewer items. The three scale domains align with factors identified in the literature as having influence on contraceptive use in SSA [10–12]. Notably, items pertaining to the fourth hypothesized domain of “friend/family influence” were not retained in statistical analyses, indicating that social influence may be less relevant to CSE in these settings. Further research is warranted, such as cognitive interviewing to assess the response process for these items. Establishing scale reliability and validity in two distinct settings is a strength of this study. However, given that the “choosing and managing a method” domain differed for the two settings (generating two versions of a similar subscale), each should be separately tested in other contexts with similar levels of contraceptive prevalence. For example, the three-item subscale which we tested in Nigeria should be assessed to see if it is reliable and valid in other low-mCPR settings, while the four-item subscale that we tested in Kenya should be similarly assessed in higher-mCPR settings.

In assessment of criterion-related validity, the AUC in Kenya for quartiles of total score fell just above our cutoff point (0.60), while the AUC of continuous total score and the total of each subscale fell below. These findings indicate either that current modern contraceptive use may not be an appropriate “gold standard” measure for CSE or that CSE may not serve in a predictive capacity for modern contraceptive use in this context. In contrast, validity results were consistently strong in Nigeria. Regression results show a strong relationship between CSESSA score and current modern contraceptive use in both settings.

The CSESSA scale and subscales can be used in contraceptive research and programming to pinpoint key intervention opportunities and to evaluate program effectiveness. Assessing mean scores by subscale and demographics, as we demonstrate in Table 3, can help to identify those who need contraceptive care support. For example, our findings indicate that women with lower education in Nigeria may need more support to communicate with their husband/partner about contraception. Our findings related to influence of age [[24], [25], [26]], education [11,26] and wealth [25,26] on CSE are consistent with literature on these factors' influence on contraceptive uptake (Table 3). Mean scores were higher in the Kenyan as compared to the Nigerian sample, reflecting differences in norms of contraceptive use and prevalence between the two settings. Additional research should be done to assess how prior contraceptive use influences women's CSE and the measure's relation to contraceptive discontinuation.

The mCPR in both sample populations is higher than national estimates (Kenya: 73.3% vs. 53.0%; Nigeria: 27.5% vs. 12.0% nationally) [14,15]. Given the eligibility criteria for the parent study, these samples may represent a specific subset of women who have higher care-seeking behavior, likelihood of using contraception and CSE compared to women who were not recruited.

This study has several limitations. First is the fact that the scale was included in a postpartum survey resulting in a homogeneous sample in terms of variation in reproductive experiences, health system interactions, preferences and partner status. Items pertaining to husband/partner communication may be less relevant to women at various stages of life and in different settings. This homogeneity may contribute to high IICs, particularly in Nigeria. Also, the cross-sectional nature of administration is not ideal. Preferably, the scale would be administered at multiple time points to gauge whether levels of CSE fluctuate or hold constant relative to an intervention.

Lastly, in the Kenyan sample, distribution is heavily skewed toward high scores. This likely is a reflection of Kenya's high mCPR; however, it is also possible that social desirability bias or survey fatigue contributed to consistently high scores. The CSESSA scale appears to be a reliable, valid measure of CSE for women in both Kenya and Nigeria.

## Appendix Supplementary data

Supplementary material Image 1

## References

[1] Bandura A. (1986). The explanatory and predictive scope of self-efficacy theory. J Soc Clin Psychol..

[2] Levinson R.A., Wan C.K., Beamer L.J. (1998). The Contraceptive Self-Efficacy Scale: analysis in four samples. J. Youth Adolesc.

[3] Arias M.L.F., Champion J.D., Soto N.E.S. (2017). Adaptation of the contraceptive self-efficacy scale for heterosexual Mexican men and women of reproductive age. Appl Nurs Res.

[4] Peyman N., Hidarnia A., Ghofranipour F., Kazemnezhad A., Oakley D. (2009). Self-efficacy: does it predict the effectiveness of contraceptive use in Iranian women?. East Mediterr Health J.

[5] Heinrich L.B. (1993). Contraceptive self-efficacy in college women. J Adolesc Health..

[6] Brafford L.J., Beck K.H. (1991). Development and validation of a condom self-efficacy scale for college students. J Am Coll Health.

[7] Farmer M.A., Meston C.M. (2006). Predictors of condom use self-efficacy in an ethnically diverse university sample. Arch Sex Behav.

[8] Quinn-Nilas C., Milhausen R.R., Breuer R., Bailey J., Pavlou M., DiClemente R. (2016). Validation of the sexual communication self-efficacy scale. Health Educ Behav.

[9] Cecil H., Pinkerton S.D. (1998). Reliability and validity of a self-efficacy instrument for protective sexual behaviors. J Am Coll Health.

[10] Cleland J., Shah I.H., Benova L. (2015). A fresh look at the level of unmet need for family planning in the postpartum period, its causes and program implications. Int Perspect Sex Reprod Health..

[11] Oladapo O.T., Iyaniwura C.A., Sule-Odu A.O. (2008). Quality of antenatal services at the primary care level in southwest Nigeria. Afr J Reprod Health.

[12] Adegbola O., Okunowo A. (2009). Intended postpartum contraceptive use among pregnant and puerperal women at a university teaching hospital. Arch Gynecol Obstet.

[13] (2015). Kenya National Bureau of Statistics, Ministry of Health/Kenya, National AIDS Control Council/Kenya, Kenya Medical Research Institute, National Council for Population and Development/Kenya.

[14] National Population Commission - — NPC and ICF (2019). Nigeria demographic and health survey 2018 — final report.

[15] Kabue M.M., Grenier L., Suhowatsky S., Oyetunji J., Ugwa E., Onguti B. (2018). Group versus individual antenatal and first year postpartum care: study protocol for a multi-country cluster randomized controlled trial in Kenya and Nigeria. Gates Open Res.

[16] Rutstein S.O. (2019). teps to constructing the new DHS Wealth Index. https://www.dhsprogram.com/topics/wealth-index/Wealth-Index-Construction.cfm.

[17] Nunnally J.C., Wolman B.B. (1978). An overview of psychological measurement. Clinical diagnosis of mental disorders.

[18] Zijlmans E.A.O., Tijmstra J., van der Ark L.A., Sijtsma K. (2018). Item-score reliability in empirical-data sets and its relationship with other item indices. Educ Psychol Meas.

[19] Matsunaga M. (2010). How to factor-analyze your data right: do’s. don’ts, and how. Int J Psychol Res (Medellin).

[20] Crawford A.V., Green S.B., Levy R., Lo W.J., Scott L., Svetina D. (2010). Evaluation of parallel analysis methods for determining the number of factors. Educ Psychol Meas.

[21] DeVellis R. (2003). Scale development: theory and applications. applied social methods research series vol. 26.

[22] Park S.H., Goo J.M., Jo C.H. (2004). Receiver operating characteristic (ROC) curve: practical review for radiologists. Korean J Radiol.

[23] Zeger S.L., Liang K.Y. (1986). Longitudinal data analysis for discrete and continuous outcomes. Biometrics.

[24] Idowu A., Deji S.A., Ogunlaja O., Olajide S.O. (2015). Determinants of intention to use post partum family planning among women attending immunization clinic of a tertiary hospital in Nigeria. Am J Public Health Res.

[25] Akinlo A., Bisiriyu A., Esimai O. (2013). Use of maternal health care as a predictor of postpartum contraception in Nigeria. Etude Popul Afr.

[26] Hounton S., Winfrey W., Barros A.J.D., Askew I. (2015). Patterns and trends of postpartum family planning in Ethiopia, Malawi, and Nigeria: evidence of missed opportunities for integration. Glob Health Action.

