# Supplementary material for: Measuring contraceptive self-efficacy in sub-Saharan Africa: development and validation of the CSESSA scale in Kenya and Nigeria
Source: Contracept X. 2020 Oct 9;2:100041. doi: 10.1016/j.conx.2020.100041 (PMC7591732; doi:10.1016/j.conx.2020.100041)
Supplement: Supplementary file 1 — Supplementary material [file mmc1.pdf]

Appendix A

Original 18 item Scale

1. Discuss family size with my husband/partner
2. Discuss if and when I'd like to get pregnant again with my husband/partner
3. Discuss specific family planning methods with my husband/partner
4. Ask my husband/partner to use a condom if I want him to
5. Reach an agreement with my husband/partner about use of family planning that takes my desires into account
6. Start a family planning method if my friends and family might find out
7. Continue a family planning method if my friends and family found out
8. Bring up the topic of family planning with a health care provider
9. Ask a provider questions I have about family planning methods
10. Ask a provider to clarify something they have told me about family planning if I'm not sure I understand
11. Tell a provider what's important to me in choosing a family planning method
12. Have some control over if and when I get pregnant again
13. Choose a family planning method that will work well for me
14. Obtain the method of family planning I want, if I want one
15. Obtain a different method of family planning if the one I want isn't available
16. Find solutions to bothersome side effects from family planning or switch methods if needed because of bothersome side effects
17. Use a family planning method according to instructions to prevent pregnancy
18. Stop using family planning and get pregnant again if/when I want to

Appendix B

The CSESSA scale comprises of three sub-scales: Self-efficacy for husband/partner communication on family planning, Self-efficacy for provider communication on family planning, and Self-efficacy for choosing and managing a method of family planning. The latter sub-scale has two options for the researcher to select from depending on the contraceptive prevalence of their study setting. When measuring contraceptive self-efficacy, all three sub-scales are to be used together in the following order.

Self-efficacy for husband/partner communication on family planning

1. Discuss family size with my husband/partner
2. Discuss if and when I'd like to get pregnant again with my husband/partner
3. Discuss specific family planning methods with my husband/partner
4. Reach an agreement with my husband/partner about use of family planning that takes my desires into account

Self-efficacy for provider communication on family planning

1. Bring up the topic of family planning with a health care provider
2. Ask a provider to clarify something they have told me about family planning if I'm not sure I understand
3. Tell a provider what's important to me in choosing a family planning method

Self-efficacy for choosing and managing a method of family planning (choose appropriate sub-scale from below)

Use in a low mCPR setting

1. Obtain the method of family planning I want, if I want one
2. Obtain a different method of family planning if the one I want isn't available
3. Stop using family planning and get pregnant again if/when I want to

Use in a high mCPR setting

1. Choose a family planning method that will work well for me
2. Obtain the method of family planning I want, if I want one
3. Find solutions to bothersome side effects from family planning or switch methods if needed because of bothersome side effects
4. Use a family planning method according to instructions to prevent pregnancy

Appendix C

Details on validation methods

Criterion-related validity, which represents the empirical association between the scale and an external criterion, often referred to as the "gold standard" [13] was explored via the area under the curve (AUC) of a receiver operating characteristic (ROC) curve. The AUC is a summary measure of diagnostic performance ranging from 0 to 1 and provides the average sensitivity value for all possible specificity values. As the AUC approaches 1, the diagnostic performance of a test improves [14]. We calculated the AUC for a ROC to assess the predictive capability of the mean CSESSA score against current modern contraceptive use. Criterion-related validity was considered to be established if the AUC was greater than 0.60. Construct validity, the extent to which a measure performs as one would anticipate in relation to established measures of other related constructs [13], was assessed through logistic regression of the total CSESSA score against current modern contraceptive use.

If we were to strictly assess the scale by language, the subject-to-item ratio allows for scale validation in Kiswahili and Luo in Kenya, and Hausa in Nigeria. From this standpoint, sample size is lacking in English (both sites) and Kamba (Kenya), although findings signal that the scale works well consistently across these languages. Results from multivariate regression in both settings showed participant language to be non-significant, indicating that a woman's score was associated with modern contraceptive use regardless of language.

Appendix D

Rotated Factor Loadings* for CSESSA Scale Items by domain: Kenya and Nigeria

	**Kenya n=314**				**Nigeria n=414**			
Item	Husband/partner communication	Provider communication	Choosing and managing a method	Uniqueness	Husband/partner communication	Provider communication	Choosing and managing a method	Uniqueness
Discuss family size with my husband/partner	0.90	-0.07	0.02	0.23	0.78	0.06	0.04	0.31
Discuss if and when I'd like to get pregnant again with my husband/partner	0.90	0.01	-0.09	0.24	0.82	0.03	0.03	0.28
Discuss specific family planning methods with my husband/partner	0.74	0.09	0.02	0.37	0.96	-0.01	-0.03	0.11
Reach an agreement with my husband/partner about use of family planning that takes my desires into account	0.66	0.04	0.14	0.42	0.91	-0.03	0.05	0.15
Bring up the topic of family planning with a health care provider	0.00	0.80	0.03	0.33	0.03	0.86	0.06	0.18
Ask a provider to clarify something they have told me about family planning if I'm not sure I understand	-0.01	0.77	0.17	0.22	-0.01	0.99	-0.02	0.05
Tell a provider what's important to me in choosing a family planning method	0.02	0.82	0.02	0.29	-0.00	0.93	0.00	0.14
Choose a family planning method that will work well for me	0.00	0.06	0.79	0.31	-	-	-	-
Obtain the method of family planning I want, if I want one	-0.04	0.06	0.86	0.23	0.20	0.07	0.68	0.27
Obtain a different method of family planning if the one I want isn't available	-	-	-	-	-0.01	0.01	0.97	0.06
Find solutions to bothersome side effects from family planning or switch methods if needed because of bothersome side effects	0.06	0.08	0.75	0.29	-	-	-	-
Use a family planning method according to instructions to prevent pregnancy	0.01	-0.01	0.85	0.28	-	-	-	-
Stop using family planning and get pregnant again if/when I want to	-	-	-	-	0.00	-0.01	0.93	0.15

*Factor loadings indicate the structure of the scale. We used the following criteria to categorize items into three factors/domains based on the data shown: items were retained if their greatest factor loading was >0.60 and second highest was <0.30, items with uniqueness >0.50 were to be removed. Domain names were attributed to factors based on the content of their retained items.
